# Leveraging Spatial Variation in Tumor Purity for Improved Somatic Variant Calling of Archival Tumor Only Samples

**DOI:** 10.3389/fonc.2019.00119

**Published:** 2019-03-20

**Authors:** Rebecca F. Halperin, Winnie S. Liang, Sidharth Kulkarni, Erica E. Tassone, Jonathan Adkins, Daniel Enriquez, Nhan L. Tran, Nicole C. Hank, James Newell, Chinnappa Kodira, Ronald Korn, Michael E. Berens, Seungchan Kim, Sara A. Byron

**Affiliations:** ^1^Quantitative Medicine and Systems Biology Division, Translational Genomics Research Institute, Phoenix, AZ, United States; ^2^Integrated Cancer Genomics Division, Translational Genomics Research Institute, Phoenix, AZ, United States; ^3^Mayo Clinic, Scottsdale, AZ, United States; ^4^Imaging Endpoints, Scottsdale, AZ, United States; ^5^HonorHealth Scottsdale Shea Medical Center, Scottsdale, AZ, United States; ^6^GE Global Research Center, Niskayuna, NY, United States; ^7^PureTech Health, Boston, MA, United States; ^8^Cancer and Cell Biology Division, Translational Genomics Research Institute, Phoenix, AZ, United States; ^9^Prairie View A&M University, Prairie View, TX, United States

**Keywords:** cancer genomics, somatic variant calling, next generation sequencing, tumor-only sequencing, tumor exome sequencing, cancer hotspot mutations

## Abstract

Archival tumor samples represent a rich resource of annotated specimens for translational genomics research. However, standard variant calling approaches require a matched normal sample from the same individual, which is often not available in the retrospective setting, making it difficult to distinguish between true somatic variants and individual-specific germline variants. Archival sections often contain adjacent normal tissue, but this tissue can include infiltrating tumor cells. As existing comparative somatic variant callers are designed to exclude variants present in the normal sample, a novel approach is required to leverage adjacent normal tissue with infiltrating tumor cells for somatic variant calling. Here we present lumosVar 2.0, a software package designed to jointly analyze multiple samples from the same patient, built upon our previous single sample tumor only variant caller lumosVar 1.0. The approach assumes that the allelic fraction of somatic variants and germline variants follow different patterns as tumor content and copy number state change. lumosVar 2.0 estimates allele specific copy number and tumor sample fractions from the data, and uses a to model to determine expected allelic fractions for somatic and germline variants and to classify variants accordingly. To evaluate the utility of lumosVar 2.0 to jointly call somatic variants with tumor and adjacent normal samples, we used a glioblastoma dataset with matched high and low tumor content and germline whole exome sequencing data (for true somatic variants) available for each patient. Both sensitivity and positive predictive value were improved when analyzing the high tumor and low tumor samples jointly compared to analyzing the samples individually or *in-silico* pooling of the two samples. Finally, we applied this approach to a set of breast and prostate archival tumor samples for which tumor blocks containing adjacent normal tissue were available for sequencing. Joint analysis using lumosVar 2.0 detected several variants, including known cancer hotspot mutations that were not detected by standard somatic variant calling tools using the adjacent tissue as presumed normal reference. Together, these results demonstrate the utility of leveraging paired tissue samples to improve somatic variant calling when a constitutional sample is not available.

## Introduction

Somatic mutations often drive cancer initiation and progression. The identification of somatic mutations through next generation sequencing has enabled the identification of cancer driver events in individual patient tumor samples (1–4). There is also ongoing effort to discover new cancer driver mutations, particularly in non-coding regions (5). Although sequencing of tumor-associated cancer gene panels and exomes is starting to be adopted in clinical practice to personalize therapy, there is much to learn about how mutation status correlates with response to therapy. Clinically annotated archival tissue collections represent a rich resource for identifying new driver mutations and clarifying how genomic features relate to clinical outcomes (6, 7). There are a number of sophisticated approaches for distinguishing driver from passenger mutations, but they all require accurate variant calls as inputs (8). In order to accurately distinguish somatic from germline variant, it is important to have a matched constitutional sample. However, most archival collections do not contain blood samples or other normal tissue samples from locations distant to the tumor to use as a constitutional reference. Here we present a novel approach to get more accurate somatic variant calls from archival samples.

Often, histologically normal tissue is available alongside the tumor biopsy or resection. For instance, surgeons typically remove a margin of adjacent normal tissue when resecting a tumor. This normal tissue can be leveraged for DNA sequencing to identify germline variants. However, histologically normal tissue may still have detectable molecular alterations for a variety of reasons. For example, it is difficult to know if the adjacent normal tissue is truly free of infiltrating tumor cells. Contamination of the adjacent normal tissue with the tumor tissue during processing could also confound interpretation of the results (9). Also, even without infiltrating tumor cells, the adjacent tissue may contain somatic mutations. Field cancerization, where molecular alterations are observed in tissue adjacent to the overt cancer, is thought to be an important risk factor for multifocal and recurrent disease (10). This phenomenon has been observed in many cancer types including breast (11) and prostate (12). Even healthy individuals have somatic mutations in normal tissues, and the mutation patterns tend to be similar to those of the cancers arising from that tissue type (13). Clonal hematopoiesis represents a well-documented example of somatic mutations in a normal tissue. There even appears to be positive selection for cancer driver mutations in normal skin (14). Therefore, it is important to consider potential sources of somatic variant contamination when normal tumor-adjacent tissue is used to identify tumor specific somatic variants.

**Figure 1:**
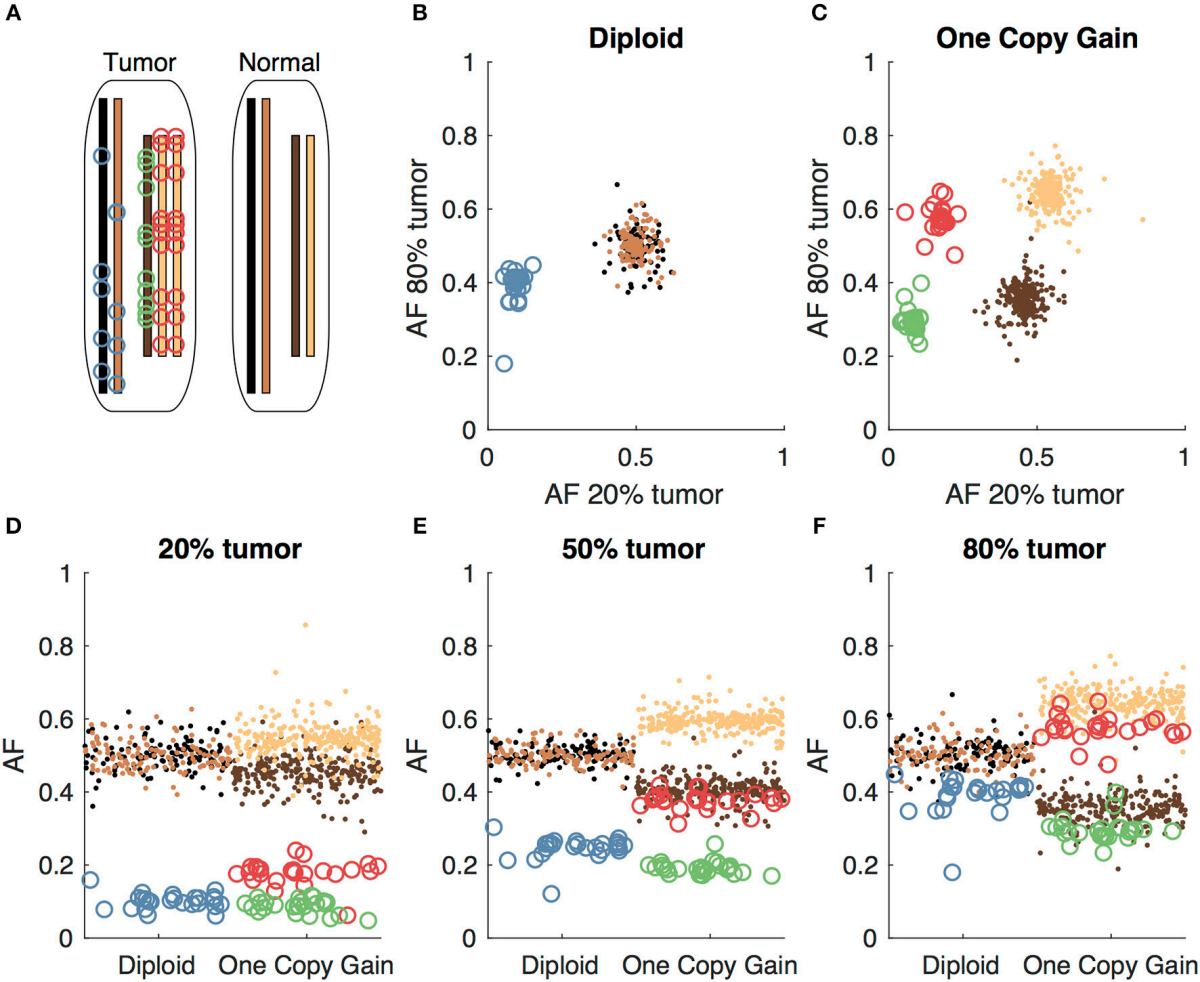
Somatic and germline variant allelic fractions example. **(A)** Two chromosomes are illustrated for this example. Both chromosomes are present in the diploid state in the normal cell. In the tumor cell, one chromosome is in the diploid state, and the other shows one-copy gain. Blue circles represent somatic variants on the diploid chromosome, green and red circles represent somatic variants on the minor and major alleles of the gained chromosome, respectively. Simulated allelic fractions of germline variants (brown/tan) and somatic variants are plotted for a simulated 20% tumor **(D)**, 50% tumor **(E)** and 80% tumor **(F)** by chromosome position. In the 50% tumor example, somatic variants could easily be distinguished from germline on the diploid chromosome, but on the copy number gain chromosome, the allelic fractions of the somatic variants on the major allele overlap with the germline variants. By using both the 20 and 80% tumor samples, the somatic variants can be separated from the germline variants by allelic fraction on both the diploid chromosome **(B)** and the copy number gain chromosome **(C)**.

When tumor-only sequencing data is available, researchers have developed various analytic strategies to distinguish germline and somatic variants. One obvious first step to identify somatic variants in tumor-only sequencing data is to filter out the germline variants found in population databases. Jones et al. showed that filtering alone is not sufficient, as each individual typically has an average of 249 private germline variants not found in the population databases that would be incorrectly classified as somatic in tumor-only sequencing (15). The number of private germline variants will vary based on the individual's ancestry. The private variant rate in a population depends both on how well-represented the population is in large scale sequencing projects, as well as the extent to which the population has undergone a recent expansion adding to the diversity of variants (16). More recently, Kalatskaya et al. published a machine-learning approach (ISOWN) to classify somatic and germline variants from tumor-only sequencing data (17). Their approach requires a large training set, and performs best when the training and test datasets are from the same cancer type and patient cohort. In the case of rare cancer types and case studies, obtaining such training sets may not be practical. The variant allele fraction, which is the fraction of reads supporting the mutated allele at a given locus, can also help to distinguish somatic from germline variants in impure tumors; the somatic variants should only be present in the tumor cells, leading to a low variant allele fraction, while the germline variants would be present in both the tumor and normal cells in the sample, leading to a variant allele fraction close to 0.5 for heterozygous variants. We, along with several other groups, have previously described methods to use the variant allele fractions to distinguish somatic and germline variants including somVarIUS (18), PureCN (19), and lumosVar 1.0. SomVarIUS assumes that variants with similar allele fractions to common germline variants within a copy number segment are germline and those with significantly different allelic fractions are somatic. Both PureCN and lumosVar 1.0 are conceptually similar in that they explicitly model integer copy number states and used the expected allelic fractions of somatic and germline variants to calculate likelihoods that variants are somatic or germline, though they differ in many of the model details. PureCN explicitly models tumor purity using the copy number and germline variant allele fractions, treating sub-clonal copy number alterations as an exception to the model. The lumosVar 1.0 model finds groups of both copy number alterations and somatic mutations that appear to occur in the same fraction of cells in the sample, thus treating sub-clonal variants more explicitly and allowing somatic mutations to inform the estimate of tumor purity. PureCN requires mutation calls as input and only removes variants that do not appear diploid in a set of unmatched normal, while lumosVar 1.0 does its own variant calling and quality filtering, taking into account both unmatched normal data and the tumor itself. A major limitation that all of these approaches have in common is that some combinations of copy number alterations and tumor purity can lead to considerable overlap in the expected somatic and germline variant allele fractions and greatly reduce the power to detect somatic variants (Figure 1). Thus, there is a need for new bioinformatics methods to call germline and somatic variants from tumor samples with high sensitivity and precision, even in the absence of a germline sample.

Here, we present a new bioinformatics approach (lumosVar 2.0) that leverages adjacent normal tissue from tumor biopsies and permits somatic mutations to be present in the adjacent tissue. Similar to our previous approach (lumosVar 1.0—single sample-based variant caller) (16), we model allelic copy number to determine the expected allelic fractions for somatic and germline variants as well as incorporate population database frequencies to call variants as somatic or germline. We have extended the approach to find the joint probability of somatic and germline mutations across multiple samples from the same patient. We hypothesize that the patterns of allelic fractions across samples of different purities will be more informative than any individual sample in distinguishing somatic from germline variants (Figure 1). To test this hypothesis, we compare two approaches (1) jointly calling variants using two samples of different purities (joint approach) and (2) pooling the two samples resulting in one sample with twice the sequencing depth and the average of the purities (pooled approach). First, we used simulations to systematically evaluate the effects of tumor purity and copy number states for the two approaches. Next, we looked at a set of glioblastoma (GBM) patient samples where we have sequencing data for contrast-enhancing region (CE, high fraction of tumor cells) and non-enhancing region (NE, low fraction of tumor cells) biopsies, as well as sequencing data from peripheral blood samples to establish true somatic calls. Finally, we applied our method to an archival cohort of breast and prostate samples where FFPE sections from the tumor biopsies or resections were the only tissues available.

## Methods

### Simulations

We used simulated read count data to systematically determine how the purity of the two tumor samples, the copy number, and the read depth affect our ability to detect somatic variants. The simulations were performed as previously described (16), where the total read depths were drawn from a log normal distribution and the number of reads supporting the variant were drawn from a binomial distribution with a probability of success of the expected allelic fraction given the tumor purity and copy number state. We simulated 1,000 somatic variants and 10,000 germline heterozygous variants for each coverage level and copy number state. To evaluate how the joint calling approach compares to the single sample approach, we added the read depths of each pair of simulated tumor samples used jointly.

### Evaluation Dataset

A set of previously collected and de-identified whole exome data from seven recurrent GBM patients was used to evaluate the approach (Table 1). Each patient dataset contained exome sequencing data for CE biopsies (high tumor content), NE biopsies (low tumor content), and peripheral blood (germline). The acquisition and sequencing of these samples was performed following IRB approval and patient informed consent, as previously described (20). The consensus of three comparative somatic variant callers [seurat (21), strelka (22), and mutect (23)] using the CE samples as tumor and the peripheral blood as normal was used to define the true somatic variants. Variants called by only one of the three somatic variant callers were not counted as true positives or true negatives in the evaluation. Since lumosVar 2.0 could call variants that were only found in the NE samples, but that was not the goal of the evaluation, we also ran the three paired somatic variant callers using the NE samples as the tumor and the blood as the normal, and excluded variants detected in only the NE samples from the evaluation. In order to evaluate the benefit of jointly calling high tumor and low tumor content samples compared to our prior single sample tumor only approach, we merged the bam files from the CE and NE samples, and also called variants on these merged bams using lumosVar 2.0. We call the lumosVar 2.0 analysis of the merged CE and NE bams the pooled approach. In order to compare our results to what one would expect from using germline snp databases to classify variants as somatic or germline, we used dbSNPv149 (24), after excluding snps where the allele of origin was annotated as somatic. We determined the number of likely germline false positives based on the number of heterozygous variants called by GATK HaplotypeCaller (25) that were not found in the somatic excluded dbSNP set, and the number of somatic false negatives based on the number of true somatic variants found in the somatic excluded dbSNP set.

**Table 1:** Patient characteristics.

			**Mean target coverage**		
**Patient Id**	**Cancer type**	**Low tumor source^a^**	**Low tumor**	**High tumor**	**Peripheral blood**
GBM-003	GBM	NEB	183	387	177
GBM-005	GBM	NEB	482	404	144
GBM-006	GBM	NEB	441	248	115
GBM-008	GBM	NEB	203	424	186
GBM-009	GBM	NEB	199	387	153
GBM-014	GBM	NEB	223	366	114
GBM-016	GBM	NEB	416	376	261
BHH01	Breast	ANWS	255	268	NA
BHH02	Breast	ANMD	296	350	NA
BHH03	Breast	ANMD	228	302	NA
BHH04	Breast	ANMD	269	296	NA
BHH06	Breast	ANMD	249	336	NA
BHH09	Breast	ANMD	312	290	NA
BHH11	Breast	ANMD	249	282	NA
BHH15	Breast	ANWS	211	323	NA
BHH16	Breast	ANWS	294	289	NA
BHH21	Breast	ANMD	286	331	NA
BHH22	Breast	ANWS	226	301	NA
BHH24	Breast	ANWS	229	297	NA
BHH25	Breast	ANWS	278	275	NA
BHH26	Breast	ANMD	301	291	NA
BHH27	Breast	ANWS	236	328	NA
BHH28	Breast	ANWS	275	264	NA
BHH08	Breast	ANWS	286	288	NA
BHH18	Breast	ANWS	287	261	NA
BHH20	Breast	ANWS	185	326	NA
BHH23	Breast	ANWS	273	243	NA
HHP01	Prostate	ANWS	202	216	NA
HHP02	Prostate	ANWS	206	221	NA
HHP03	Prostate	ANWS	261	184	NA
HHP04	Prostate	ANWS	312	241	NA
HHP05	Prostate	ANWS	247	232	NA
HHP06	Prostate	ANWS	294	214	NA
HHP07	Prostate	ANWS	265	254	NA
HHP08	Prostate	ANWS	287	247	NA
HHP09	Prostate	ANWS	302	228	NA
HHP10	Prostate	ANWS	328	277	NA
HHP11	Prostate	ANWS	329	274	NA
HHP12	Prostate	ANWS	238	239	NA
HHP13	Prostate	ANWS	299	285	NA
HHP14	Prostate	ANWS	269	269	NA
HHP16	Prostate	ANWS	363	330	NA
HHP17	Prostate	ANWS	255	316	NA
HHP18	Prostate	ANWS	224	258	NA
HHP19	Prostate	ANWS	241	281	NA
HHP20	Prostate	ANWS	208	307	NA
HHP21	Prostate	ANWS	213	263	NA

a *NEB, non-enhancing biopsy; ANWS, adjacent normal whole slide; ANMD, adjacent normal macrodissected*.

### Application to Archival Sample Sets

De-identified FFPE tissue sections, clinical data, and pathology data were acquired for 20 breast cancer patients and 20 prostate cancer patients from HonorHealth Scottsdale Shea Medical Center, in accordance with local institutional review boards and in compliance with the Health Insurance Portability and Accountability Act (HIPAA) (Table 1). Prostate cancer specimens were collected under IRB approved protocol with 45 CFR 46.111 (d) exemption; breast cancer specimens were collected under IRB approved protocol including patient informed consent per institutional policy and procedures. Retrospective analysis was performed using archival samples from treatment-naïve, invasive breast carcinomas or treatment-naïve prostate adenocarcinomas.

Breast tumors were collected following routine clinical lumpectomy or mastectomy, from women diagnosed with ER-positive, invasive mammary carcinoma between 2010 and 2016 at HonorHealth Scottsdale. Median age of diagnosis was 65 years and ranged from 39 to 86 years. All tumors were classified by pathology as estrogen receptor-positive. Nineteen of the twenty tumors were classified as HER2-negative. The breast tumor cohort spanned AJCC stages (IA-IV). Prostate tumors were collected following radical prostatectomy for men diagnosed with prostate adenocarcinoma between 2012 and 2016 at HonorHealth Scottsdale. Median age of diagnosis was 67 years, ranging from 57 to 74 years. Eighteen of the twenty tumors had a Gleason score of seven or greater. ER/PR/HER2 status (breast tumors), Gleason score (prostate tumors), histological type, tumor stage, treatment history, and clinical outcome, including progression-free survival and overall survival, were collected from medical records and the de-identified data was provided for this study. Pathology review (JN) identified a tissue block with high tumor content and a tissue block with a region considered to have low tumor content for each patient. Five 10-micron sections were provided for each sample (5 high tumor content; 5 low tumor content). The Qiagen GeneRead FFPE DNA Kit (cat# 180134) was used to isolate DNA from FFPE breast and prostate cancer tumor specimens (*N* = 80) following the manufacturer's protocol.

Exome libraries were constructed from 200 ng of DNA (DIN = 3–5) using KAPA Biosystems' Hyper Prep Kit (cat#KK8504) and the same bait set that was used in the evaluation dataset, following the manufacturer's protocols. The bait set included Agilent's SureSelectXT V5 baits plus custom content including copy number probes distributed across the entire genome, along with additional probes targeting tumor suppressor genes and genes involved in common cancer translocations to enable structural analysis. Libraries were equimolarly pooled, quantitated, and sequenced by synthesis on the Illumina HiSeq 4000 for paired 82 bp reads.

### Other Variant Calling Approaches

Two other variant calling approaches were applied to the archival samples for comparison. The first approach, called unmatched plus filtering (UPF) used the high tumor content samples as the tumor and an unmatched normal (GM12878) as the normal in the paired somatic variant callers (mutect, strelka, and seurat). dbSNP and COSMIC were used to classify variants as somatic or germline. Variants that were called by at least two of the three paired somatic variant callers (or two out of two for indels, since mutect does not call indels) and were not present in dbSNP or were present in dbSNP and also present in COSMIC were considered somatic calls in the UPF approach. In the second approach, called adjacent normal as reference (ANR), the high tumor content sample was used as the tumor sample and the adjacent normal sample was used as the normal in the paired somatic variant callers (mutect, strelka, and seurat). Variants called by at least two out of the three paired somatic variant callers were considered somatic calls in the ANR approach.

### Variant Caller Overview

**Figure 2:**
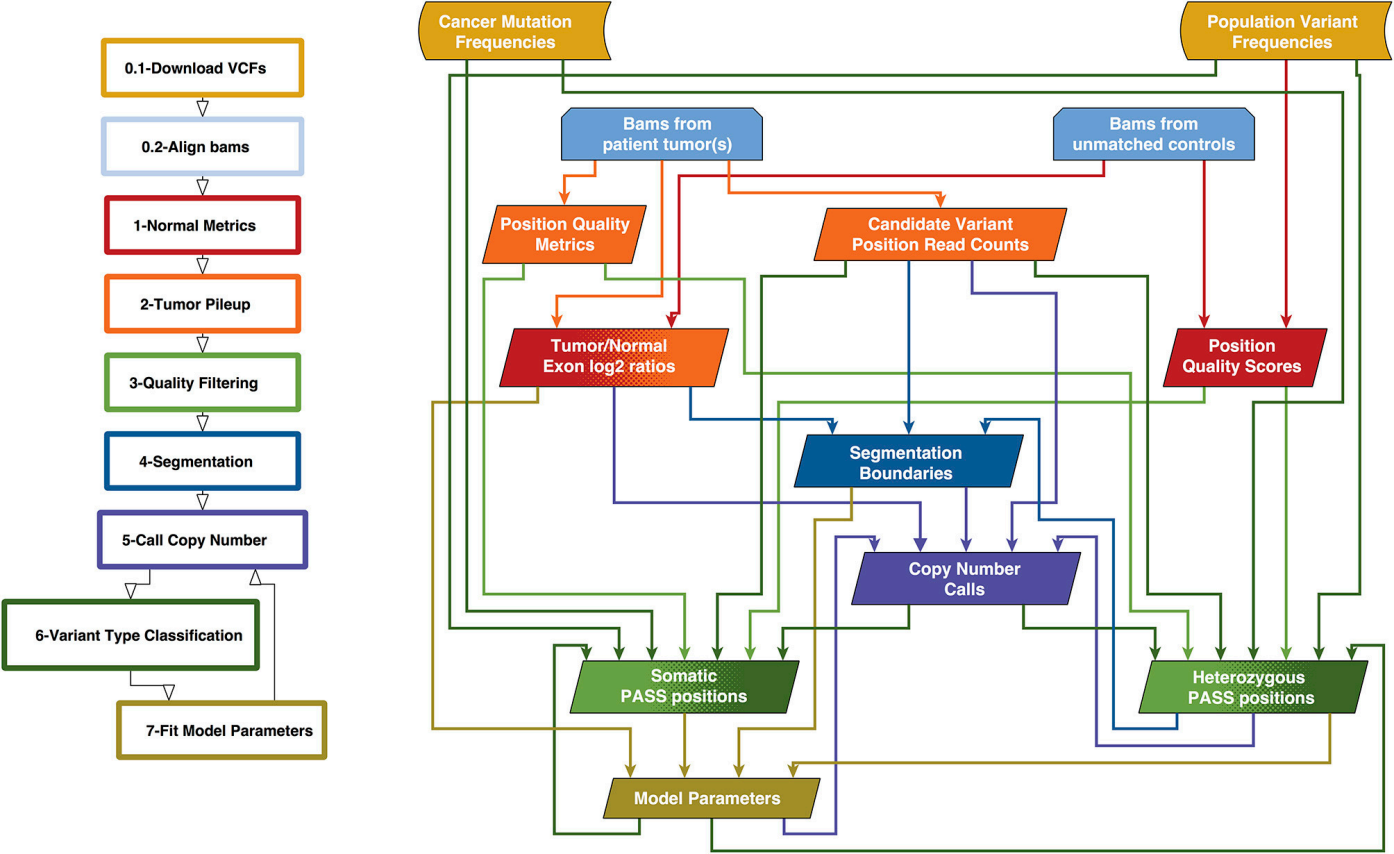
Overview of lumosVar 2.0 analysis. The flow-chart on the left show the main steps in the analysis. Steps 0.1 and 0.2 are data preparation, and steps 1–7 are performed by lumosVar 2.0. The graph on the right illustrates the main inputs and outputs of each step. The color of the arrows coming from each box indicates the steps where that data is used as input, and the color of each box indicates the step where the data is generated.

We previously created a single-sample strategy (lumosVar 1.0) to call somatic variants in impure tumor samples based on the differences in allelic frequency between the somatic and germline variants (16). Here, we describe an extension of lumosVar 1.0 to jointly analyze multiple samples from the same patient. The lumosVar 2.0 analysis has seven main steps (Figure 2). First, a set of unmatched control samples is analyzed for position quality scores and average read depth, as previously described (16). Second, read counts and quality metrics are extracted from the tumor bams. Third, quality scores are calculated for each candidate variant position. Fourth, segmentation is performed to define regions that have similar tumor/normal read depth ratios and B-allele fractions. The fifth step involves finding the most likely allele-specific copy number state for each segment. The sixth step involves classifying each candidate variant position as somatic, germline heterozygous, or homozygous. The final step entails optimization of the model parameters. The caller iterates between steps five, six, and seven until the solution converges. Model input parameters and notation are shown in Tables 2, 3.

**Table 2:** Parameters and default values.

**Parameter**	**Default value or source**	**Description**
*f ^π^*	0.1,0.7	Vector of length J of initial sample fractions. Default assumes two samples, with low and high tumor content.
*α_π_*	1.5	Determines shape of prior distribution of Δf
π (*N* = 0) …π (*N* = 3), π (*N* ≥ 5)	0.01,0.25,0.3,0.2,0.15,0.09	Copy Number Priors
π (*M* = 0), π (*M* = 1), π (*M* ≥ 2)	0.25,0.5,0.25	Minor Allele Copy Number Priors
*α_*seg*_*	1E-5	Segmentation significance cutoff
ω	COSMIC	Number of cancer variants observed at the position
*F*_A_, *F_*B*_*	1,000 Genomes and Exac	Population Allele Frequencies
ρ_SNV_, *ρ_*indel*_*	1E-5, 1E-6	Constant for calculating prior somatic
*F*_p−**SNV**_, *F*_p−**indel**_	1E-5, 1E-6	Population allele frequencies assigned to alleles not seen in input population
F_max−somatic_	2E-5	Maximum population allele frequency to be considered a possible somatic variant
$Q_{min}^{m}$	10	Minimum mapping quality to count read
$Q_{min}^{b}$	5	Minimum base quality to count base
*T _PASS_*	0.8	Minimum posterior probability of belonging to the PASS group to be called pass
*T _Somatic_*	0.8	Minimum posterior probability of variant is somatic to be called somatic
*T _Germline_*	0.8	Minimum posterior probability of variant is germline to be called germline
ξ	3	Number of parameter fitting iterations without new global minimum before stopping
λ	5	Weight of penalty for adding clonal variant group

**Table 3:** Parameters and notation.

**Variable**	**Descriptions**
**INPUTS TO MODEL**	
R_T_, R_B_	Total tumor read depth, B allele read depth
R_C_	Mean read depth of unmatched normals
π_S_, π_AB_, π_AA_	prior probability of somatic, germline heterozygous, germline homozygous variant
$Q_{A}^{m},\;Q_{B}^{m}$	Mean mapping quality of reads supporting the A or B allele
$Q_{B}^{b}$	Mean base quality of bases supporting B allele
X	Total number of exons,
Y	Number of heterozygous germline variants
Z	Number of somatic variants
G	Number of segments
K	Number of clonal variant groups
J	Number of samples from the patient
η_g_	Number of bases within the bed file in segment g
η_d_	Number of bases within the bed file with min (*F*_A_, *F*_*B*_) > F_max−somatic_
**PARAMETERS FIT IN MAXIMIZATION**	
f_jk_	fraction of cells in the sample j with the variants in clone k
C	centering parameter
W	controls the spread of the allelic fraction distributions
**INTERMEDIATE VARIABLES**	
N	total copy number
M	minor allele copy number
φ^S^, φ^G^	expected allele fraction of somatic or germline variant
I_S_, I_j_	Index of clonal subset containing somatic variant or copy number variant
A	Allele of somatic variant (A = 1 for allele A = 2 for minor allele)
X^CNA^	Number of copy number altered exons
**OTHER NOTATION**	
G_AA_, G_AB_	Germline homozygous or heterozygous genotype
O	Other genotype beside somatic, germline homozygous AA, or germline heterozygous AB
U	Unknown genotype due to poor mapping
k	Index of clonal subset {1, 2, …, K}
g	Index of segment {1, 2, …, G}
z	Index of somatic variant {1, 2, …, Z}
y	Index of heterozygous variant {1, 2, …, Y}
x	Index of exon {1, 2, …, X}

### Quality Classification and Filtering

We used 16 quality metrics in a quadratic discriminant model to determine the posterior probability that each position belongs to a PASS group, as was previously used in lumosVar 1.0. The same quality metrics and thresholds are used in lumosVar 2.0 as were used in lumosVar 1.0 to assign candidate variant positions to PASS and REJECT training groups (16). As previously, here we also fit the model separately on candidate indel and point mutation positions. Since the quality metrics for the B allele are not relevant for homozygous positions, after the joint variant calling is performed as described below, we repeat the quality classification step fitting homozygous positions separately after setting all the quality metrics to only use the “A” allele and setting the difference metrics to zero. The model is fit independently on each sample, and then we calculate a trust score by taking the geometric mean of the posterior probability of belonging to the PASS group weighted by the number of reads supporting the variant across the samples. Candidate positions with a trust score greater than a threshold (T_pass_) are considered for classification as somatic or germline variants as described in the joint somatic variant calling section below.

### Segmentation

Prior to fitting the copy number model, segmentation is performed. We use the circular binary segmentation algorithm implemented in the Matlab Bioinformatics toolbox to segment both the tumor to normal read depth log2 ratio of each exon and the B-allele frequencies of common heterozygous variants. Segmentation is performed independently on each sample. We combine all of the segmentation boundaries from all of the samples for both the read depth log2 ratio and B-allele frequency segmentation, and then remove non-significant segments as follows. A two-sample *t*-test is used to compare each pair of adjacent segments for both the read depth log2 ratios and the B-allele fractions for each sample. For each segmentation boundary, the geometric mean is used to combine the *p*-values across the samples and data types. The segmentation boundary with the highest geometric mean *p*-value is removed, and the *t*-tests are then performed on the newly merged segment with its neighbors. This process is continued until of the segmentation boundaries have geometric mean *p*-values less than the segmentation significance threshold (α _*seg*_).

### Expectation Maximization

We use an expectation maximization approach to fit the model parameters and call variants. In the initial iteration, heuristics are used to find reasonable values of the model parameters. In the expectation step, the model parameters are used to identify somatic and germline heterozygous variant positions. Identifying these variant positions involves finding the copy number states of each segment, joint variant type classification, and variant quality filtering, all as described below. Using those variant positions, values of the model parameters that maximize the likelihood of the data are found.

### Initial Parameter Values

The parameter f is a matrix with the number of rows corresponding to the number of clonal variant groups (K) and the number of columns corresponding the number of samples (J). The initial value of f is set such that there is a main clonal variant group that has a high sample fraction in all samples, there are J clonal variant groups that are clonal in each sample and low in the other samples, and there are another J clonal variant groups that are sub-clonal in each sample and very low in the other samples. The centering and spread parameters (C and W) are both vectors of length J, and their initial values are determined as previously described (16).

### Copy Number State Assignments

The copy number state of each segment (g) may be described by the total copy number (N_g_), the minor allele copy number (M_g_), and the index of clonal variant group of the segment (k_g_). The values of N_g_, M_g_, and k_g_ are found that maximize a sum of log likelihoods for the segment (SLL_g_).

$$\left\{N_{g},M_{g},\;k_{g}\right\}=\text{argmax}\left(SLL_{g}\right)$$

This sum includes the likelihoods of the exon mean read counts (L_xj_), heterozygous variant read counts (L_yj_), number of common germline variant positions that would be called germline heterozygous (*L*_*Y*_*d*_*g*_) or somatic (*L*_*Z*_*d*_*g*_), as well as the prior probabilities of the copy number states (π (N), π (M)) and sample fraction difference $\pi \left(\Delta \bar{f_{k_{g}}}\;\right)$.

$$\begin{array}{l} SLL_{g}=\sum_{j=1}^{J}(\frac{1}{X_{g}}\sum_{x=1}^{X_{g}}\log\;\left(\pi \left(N_{x}\right)L_{xj}\right)\\ \;+\frac{1}{Y_{g}}\sum_{y=1}^{Y_{g}}\log\;(\pi \left(\Delta \bar{f_{k_{g}}}\right)\pi \left(M_{x}\right)L_{yj})\\ \;+\frac{\eta_{dg}}{\eta_{d}}\log\;(L_{Y_{d}g}L_{Z_{d}g})) \end{array}$$

The likelihood calculations are defined below.

### Parameter Fitting Procedure

The values of f, W, and C are found that maximize the sum of segment log likelihoods (SLL_g_) and somatic variant log-likelihoods (L_z_). Since the number of clonal variant groups (K) changes the degrees of freedom of the model, as f is a J by K matrix, we include a penalty term for increasing K.

$$\begin{array}{l} \left\{f,W,C\right\}=\text{argmax}(\sum_{g=1}^{G}SLL_{g}+\frac{1}{Z}\sum_{z=1}^{Z}\log\left(\pi \left(\Delta \bar{f_{k}}\right)L_{z}\right)\\ \;\left(\pi \left(\Delta \bar{f_{k}}\right)L_{z}\right)-\frac{JK}{\lambda }) \end{array}$$

In order to more efficiently search the parameter space, we use the parameter values from the previous EM iteration (or the initial values in the first iteration) as the starting point for the parameter optimization. Since the heuristic used to find the initial value of C may be incorrect, particularly for higher ploidy genomes, other values of the centering parameter are also tested, and the best one is used as a starting point for optimization of all parameters. In order to find a reasonable starting point for adding an additional clonal variant group, we use the previous f matrix and test a set of random values for the additional column. We use the best one as the starting point for optimizing all of the parameters. If the maximum likelihood score improves, the procedure is repeated with an additional clonal variant group. This process continues until adding a clonal variant group fails to improve the likelihood score. Since adding a clonal variant group may make a previous clonal variant group less important to the model, we also test removing each clonal variant group, and then do another round of optimization of all parameters. If this results in a new maximum, then the procedure will be repeated removing another clonal variant group. Once removing clonal variant groups no longer improves the model, the procedure returns to re-centering. The re-centering, adding clonal variant groups, and removing clonal variant groups is repeated until there are ξ consecutive iterations with no new maximum found.

### Likelihood Calculations

As in lumosVar 1.0, the likelihood of the exon mean read depths are modeled as a Poisson distribution, and the somatic and germline heterozygous read counts are modeled as beta binomial distributions.

$$\begin{array}{l} L_{xj}\left(C,f|R_{Txj},R_{Cx}\right)\\ \;=poisson_{pdf}(\text{round}\left(R_{Txj}\right),\\ \;\text{round}\left(\frac{1}{C}\left(N_{x}f_{jk}R_{Cx}+2\;(1-f_{jk}\right)R_{Cx}\right))\\ L_{yj}\left(C,f,W|R_{Byj},R_{Tyj}\right)\\ \;={\text{betabinomial}}_{pdf}\left(R_{Byj},R_{Tyj},W\phi_{gjk}^{G},W\left(1-\phi_{gjk}^{G}\right)\right)\\ L_{zj}\left(C,f,W|R_{Bzj},R_{Tzj}\right)\\ \;={\text{betabinomial}}_{pdf}\left(R_{Bzj},R_{Tzj},W\phi_{gjk}^{S},W\left(1-\phi_{gjk}^{S}\right)\right) \end{array}$$

The expected germline heterozygous variant allele fraction is determined as follows.

$$\phi_{jg}^{G}=\;\frac{f_{jk_{g}}M_{g}+\left(1-f_{jk_{g}}\right)}{f_{jk_{g}}N_{g}+2\left(1-f_{jk_{g}}\right)}$$

lumosVar 2.0 considers three possible scenarios when finding the expected somatic variant allele fraction: (1) variant is in the same clonal variant group as the copy number alteration effecting the segment and is on the minor allele (*k*_*zj*_ ≡ *k*_*gj*_∧*A*_*z*_ ≡ 1), (2) variant is in the same clonal variant group as the copy number alteration effecting the segment and is on the major allele (*k*_*zj*_ ≡ *k*_*gj*_ ∧ *A*_*z*_ ≡ 2), or (3) variant is in a non-copy number altered clonal variant group (*k*_*zj*_ ≠ *k*_*gj*_).

$$\phi_{z}^{S}=\{\begin{array}{c} k_{zj}\equiv k_{gj}\wedge A_{z}\equiv 1,\;\left(f_{jk}M_{g}\right)/\left(f_{jk}N_{g}+2*\left(1-f_{jk}\right)\right)\;\\ k_{zj}\equiv k_{gj}\wedge A_{z}\equiv 2,\;\left(f_{jk}\left(N_{g}-M_{g}\right)\right)/\left(f_{jk}N_{g}+2*\left(1-f_{jk}\right)\right)\;\\ k_{zj}\neq k_{gj},\;\left(f_{jk_{z}}\right)/\left(f_{jk_{g}}N_{g}+2*\left(1-f_{jk_{g}}\right)\right)\; \end{array}$$

The maximum likelihood is used to determine the clonal variant group assignment and allele of each somatic variant.

$$\left\{k_{z},A_{z}\right\}=\text{argmax}\left(\sum_{j=1}^{J}L_{zj}\right)$$

The probability of detecting a heterozygous variant in each segment is calculated based on the cumulative probability of observing at least the minimum number of reads required to be considered a candidate variant position (R_B−min_), given the mean read depth in the segment (R_T_) and the expected allele fraction of a heterozygous variant in that segment ($\phi_{jg}^{G}$).

$$P_{het-jg}={\text{binomial}}_{cmf}\left(R_{\text{B}-\text{min}},R_{Tj},\phi_{jg}^{G}\right)$$

In order to determine if parameter values would result in reasonable variant counts, the variant type classification is performed at common germline variant positions. The likelihood of detecting fewer than the observed number of heterozygous variants in a segment (Y_dg_) is modeled as the cumulative probability from a binomial distribution with Y_dg_ successes, the number of bases examined in the segment (η_dg_), and *P*_*het*_ probability of success.

$$L_{Y_{d}g}\;(W,f|Y_{g})={\text{binomial}}_{cmf}\left({\text{Y}}_{dg},\eta_{dg},P_{het-jg}\right)$$

In order to penalize models that would result in germline variants being called somatic, we then determine the likelihood of finding that many or more somatic variants in germline variant positions based on the cumulative probability from a binomial distribution with Z_dg_ somatic variants detected of η_*dg*_ database variant positions tested, with a probability of success of ρ_*SNV*_.

$$L_{Z_{d}g}\;(C,\;W,f|Z_{dg})={\text{binomial}}_{cmf}\left({\text{Z}}_{dg},\eta_{dg},\rho_{SNV}\right)$$

In lumosVar 1.0, we set a prior distribution on f in order to favor models where the sample fractions are close to what is expected. In lumosVar 2.0, we set a prior distribution on the difference in f across the samples to favor models where the sample fractions differ as much or more than the expected sample fractions. The mean difference is found for prior tumor sample fractions (*f* ^π^) as follows:

$$\Delta \bar{f^{\pi }}=\frac{1}{\left(\begin{array}{c} J\\ 2 \end{array}\right)}\sum_{i=1}^{J-1}\sum_{j=i+1}^{J}abs\;(f_{i}^{\pi }-f_{j}^{\pi })+\epsilon$$

The mean difference of the sample fractions for each clone is found similarly.

$$\Delta \bar{f_{k}}=\frac{1}{\left(\begin{array}{c} J\\ 2 \end{array}\right)}\sum_{i=1}^{J-1}\sum_{j=i+1}^{J}abs\;(f_{ik}-f_{jk})+\epsilon$$

The prior probability that the sample fractions for each clone have a mean difference as much as or greater than observed is calculated from a beta distribution with a mode of the difference in the prior tumor sample fractions.

$$\pi \left(\Delta \bar{f_{k}}\right)={\text{beta}}_{\text{cdf}}\left(\Delta \bar{f_{k}},\alpha^{\pi },\frac{\alpha^{\pi }-1}{\Delta \bar{f^{\pi }}}-\alpha^{\pi }+2\right)$$

### Joint Variant Type Classification

The probability of observing the read counts in each sample (k) given that the variant is somatic (P (D_k_|S)), germline heterozygous (P (D_k_|G_AB_)), germline homozygous (P (D_k_|G_AA_)), or another genotype (P (D_k_|O)) are calculated as previously described. The prior probabilities are also calculated as previously described (16). The product of the conditional probabilities across the set of samples gives the joint probability of each variant type, given all the samples' data, as we assume that the read counts for each sample are independent. The posterior probability that a position has a somatic variant given all the samples' data is calculated as shown below.

$$\text{P}\left(\text{S|D}\right)=\frac{\prod_{j=1}^{J}\text{P}\left({\text{D}}_{j}\text{|S}\right)\pi_{\text{S}}}{\prod_{j=1}^{J}\text{P}\left({\text{D}}_{j}|{\text{G}}_{AA}\right)\pi_{AA}+\prod_{j=1}^{J}\text{P}\left({\text{D}}_{j}|{\text{G}}_{AB}\right)\pi_{AB}+\prod_{j=1}^{J}\text{P}\left({\text{D}}_{j}|\text{S}\right)\pi_{\text{S}}+\prod_{j=1}^{J}\text{P}\left({\text{D}}_{j}|\text{O}\right)\pi_{O}}$$

#### Implementation and Availability

A custom pileup engine was written in C using htslib (https://github.com/tgen/gvm). The pileup engine extracts the mean exon read depths and calculates the quality scores form the unmatched control bams, as well as extracts the read counts and quality metrics from the tumor bams. The rest of the lumosVar 2.0 analysis was written in Matlab (https://github.com/tgen/lumosVar2). A precompiled binary is provided which enables users to run lumosVar 2.0 without a Matlab license.

## Results

### Simulations: Comparison Between Pooled and Joint Approaches

**Figure 3:**
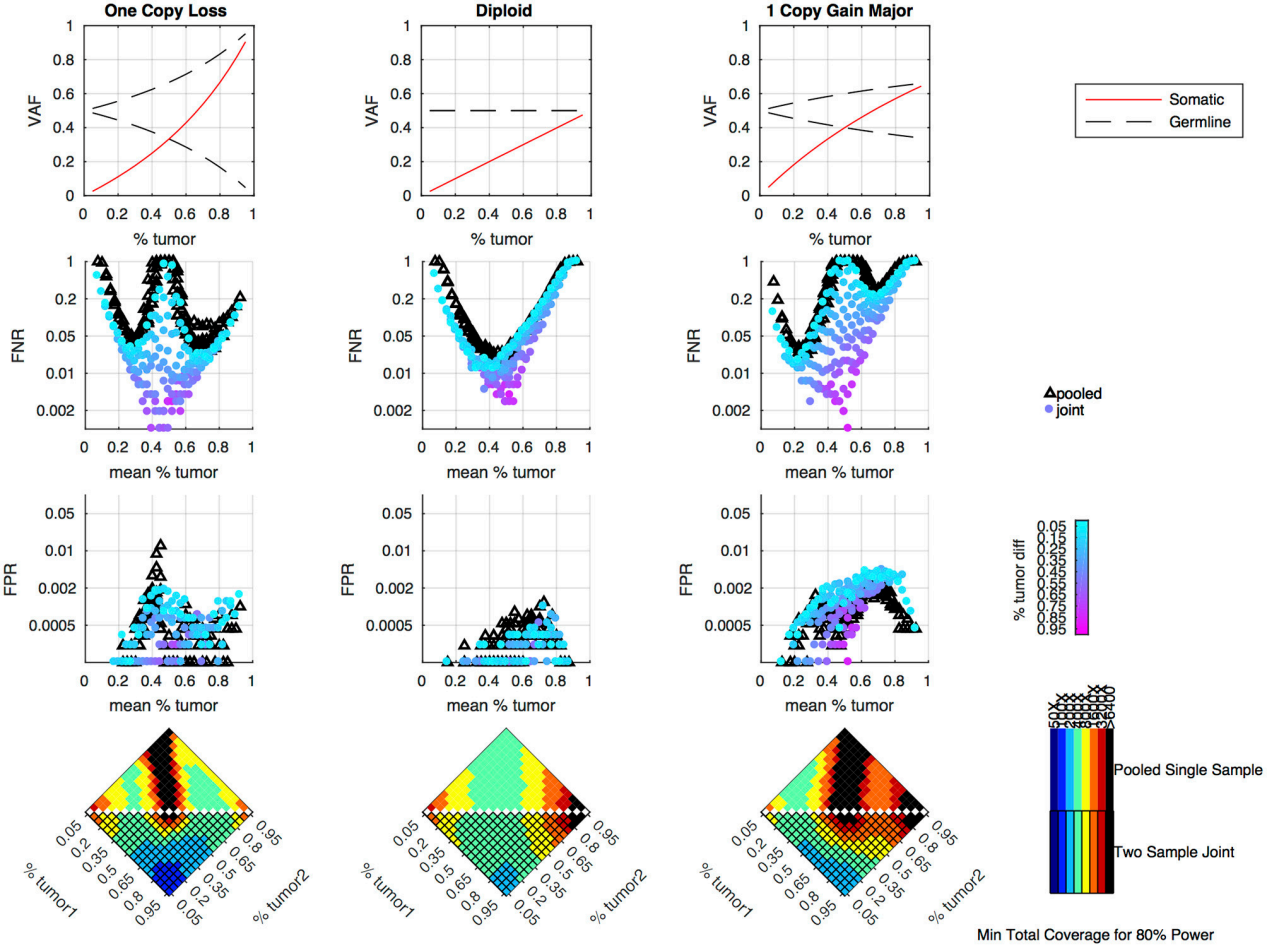
Simulation results comparing pooled and joint approaches. Top row of graphs shows the expected allele frequency of somatic (red) and germline variants (black) by tumor content (x-axis) for different copy number states. The middle two rows of graphs are based on simulation results using a mean coverage of 200X per sample (400X pooled). They show the false negative rate (FNR—simulated somatic variants not called somatic) and false positive rate (FPR—simulated germline heterozygous variants falsely called somatic) plotted by mean tumor content for the pooled (black triangles) and joint (colored circles) approaches. For the joint approach, the color of the circles represents the difference in tumor content between the two samples analyzed jointly. The bottom set of graphs shows the coverage required to detect at least 80% of the simulated somatic variants using two samples of different tumor content (shown on the x and y axis) using a joint approach (lower triangle of each heatmap) or using a single-sample approach on a merged sample with a tumor content that is the average of the two samples and coverage that is the sum of the two samples (upper triangle of heatmap). The color indicates the mean target coverage in the pooled approach, or the sum of the mean target coverage in the two-sample joint approach. Black squares indicate that <80% of the somatic variants were detected at the highest coverage simulated (6400X).

Simulations were performed to determine how the tumor purity and copy number states affect the power to detect somatic variants in the joint approach, and how the power compares to the pooled approach. As previously shown, the pooled single sample approach performs best with a sample of intermediate tumor purity for variants in diploid regions, but copy number variation leads to situations where the expected somatic and germline allele fractions are very similar, making it difficult to classify somatic variants using a single sample (16). From the simulation results, we can see that the joint approach mitigates this limitation, and only provides poor detection when both samples fall into a range where the expected somatic and germline allele fractions are very similar (Figure 3). The joint approach generally only requires low-to-moderate coverage when one sample has low tumor content and the other sample has moderate-to-high tumor content.

### Clonal Variant Groups

**Figure 4:**
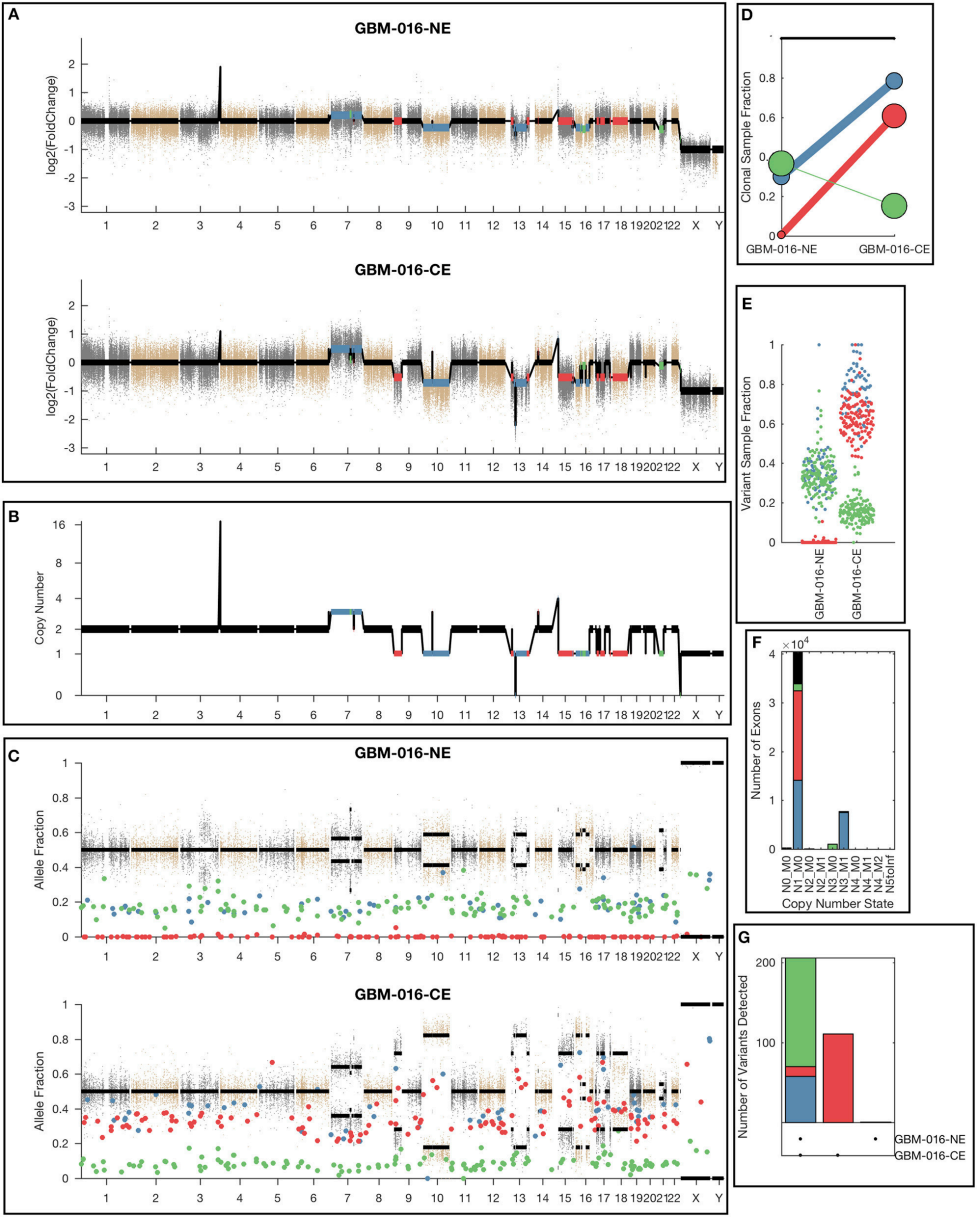
Example lumosVar 2.0 output. **(A)** Log2 fold change of the mean exon read depths compared to the unmatched controls. **(B)** The estimated integer copy number states are plotted for each genomic segment by chromosome position. **(C)** The variant allele fractions are plotted by chromosome position. The gray and brown dots represent variants called as germline heterozygous by lumosVar 2.0 and the large colored dots represent variants called somatic by lumosVar 2.0. **(D)** Summary of the clonal variant group patterns. The thickness of the lines represents the proportion of copy number events assigned to each group and the size of each circle is proportional to the number mutations assigned to each group. **(E)** Sample fraction (estimated proportion of cells in the sample containing the variant) distribution of somatic mutations. **(F)** Number of exons determined to be in each copy number state, excluding diploid. **(G)** Number of somatic mutations detected in both samples (left bar), enhancing only (middle bar), and non-enhancing only (right bar). On all plots, the colors indicate the clonal variant group.

While the single sample version of lumosVar also assigned somatic mutations and copy number alterations to clonal variant groups, these clonal variant groups become much more informative when looking at more than one patient sample. An example patient's results are shown in Figure 4. There are three clonal variant groups found in this patient, one that appears clonal in both samples (blue), one that appears sub-clonal in the enhancing biopsy and not detected in the non-enhancing biopsy (red), and one that appears clonal in the non-enhancing biopsy and sub-clonal in the enhancing biopsy (green). From these clonal variant groups, we can infer that the blue and red variants are likely found in the same cells because their sample fractions in the CE sample would add up to >100%. However, it is not possible to definitively determine from these data whether the blue and green variants are found in the same cells. The blue variants may be “trunk” mutations found in all of the tumor cells, which would imply that roughly 65% of the cells in the NE sample, and 20% of the cells in the CE sample are normal cells. It is also possible that blue and green variants are found in different sets of tumor cells, implying that roughly 35% of cells in the NE sample, and 5% of the cells in the CE sample are normal cells, highlighting the difficulty of inferring clonality and tumor evolution from a small number of tumor samples. This patient also illustrates why the joint calling approach is advantageous to detect somatic variants if the germline was not available. With only the enhancing sample, the blue variants would be difficult to differentiate from the germline variants. If the non-enhancing sample were used as a reference in standard paired somatic variant calling, only the red variants would likely be detected.

### Evaluation: Real Patients

**Figure 5:**
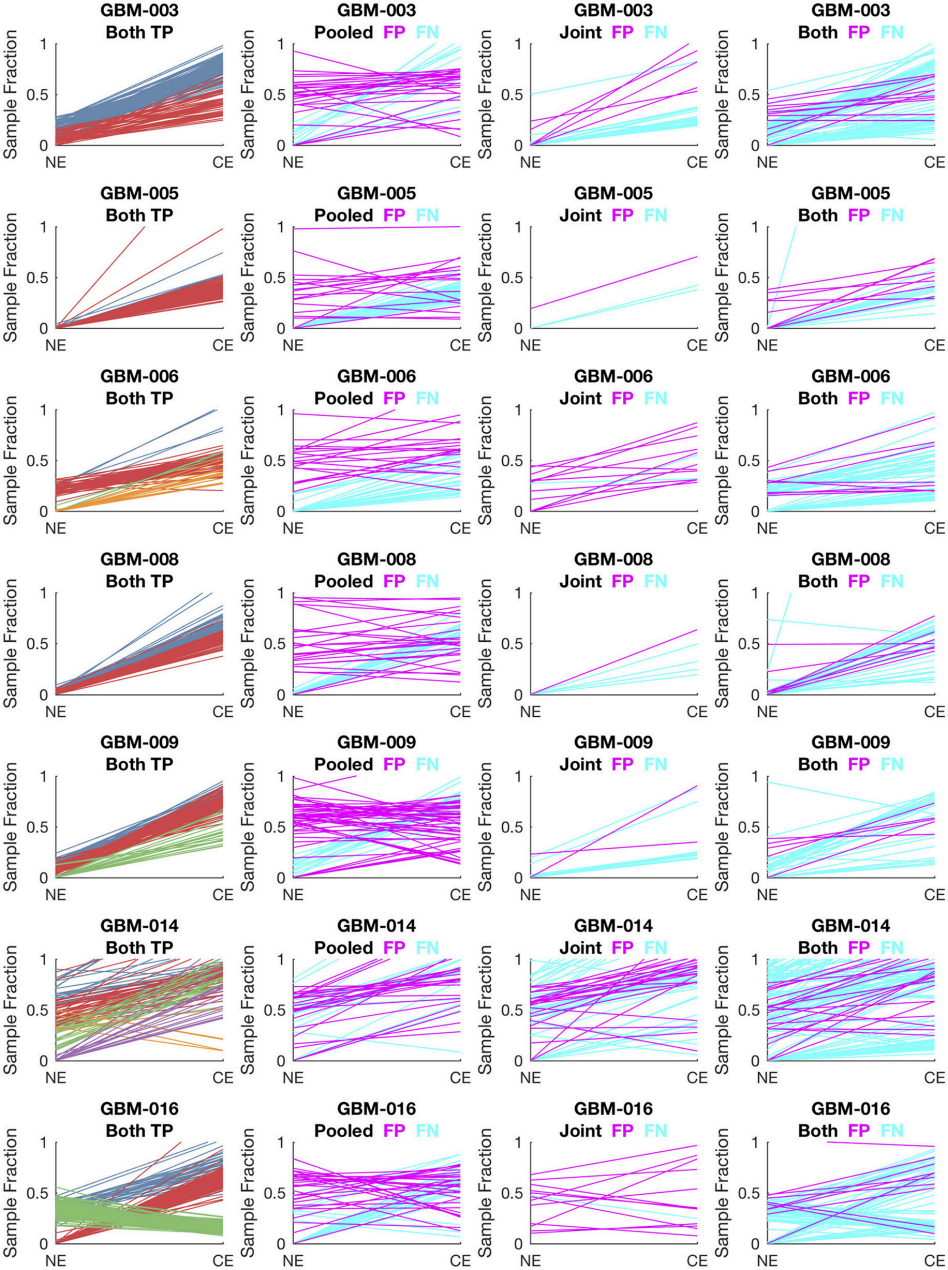
Comparison of variants called in pooled vs. joint approach. The first column of graphs shows the estimated sample fractions of true somatic variants that were detected by both the pooled and joint approaches. The variants are colored by clonal variant groups. The other three columns show the sample fractions of variants that were called incorrectly only in the pooled approach (column 2), only in the joint approach (column 3), or incorrectly in both approaches (column 4). False positives variants are shown in magenta and false negatives in cyan.

To evaluate lumosVar 2.0 on real data, recurrent glioblastoma patients that had whole exome sequencing data available for two samples of different tumor contents (from contrast enhancing and non-enhancing biopsies), as well as germline sequencing data (from peripheral blood), were identified (Table 1). Three variant calling approaches were compared: (1) A filtering approach, where heterozygous germline variants not found in dbSNP were considered false positives, and somatic variants found in dbSNP were considered false negatives, (2) a pooled approach where the data for the high tumor content and low tumor content samples are combined *in-silico*, and (3) joint analysis of the paired high tumor content and low tumor content samples. Both the pooled and joint approaches use the lumosVar 2.0 software for variant calling. We find that the filtering approach consistently has better sensitivity, but much lower precision, and lower F1 scores (harmonic mean of sensitivity and precision) than both the pooled and joint analyses (Table 4). This is consistent with our previous findings that private germline variants result in a high number of false positives using a filtering approach (16). In most of the samples, we find modest improvements in sensitivity, precision, and F1 scores in the joint approach compared to the pooled. From the simulations, we would have expected to see similar precision and more consistent improvements in sensitivity. In order to more carefully evaluate where the joint approach and pooled approach are performing differently in detecting variants, we examined the sample fractions of variants that are true positives, false positives, and false negatives in each approach (Figure 5). We find that the pooled approach has more false positive variants that have similar allelic fractions in the CE and NE biopsies. We hypothesize that these variants have unexpected allelic fractions due to mapping noise or copy number call errors that would not be modeled in the simulations. The joint approach is better at avoiding these calls, as the allelic fractions do not fit the patterns of the clonal variant groups found in the patient. However, the joint approach also misses some true somatic variants that do not fit the patterns of clonal variant groups found in the patient, such as a set of lower sample fraction variants in GBM-003. GBM-014 is the only patient where the pooled approach outperforms the joint approach. This patient also appears to have the smallest difference in tumor content between the two biopsies as well as the most complex copy number profile of this set of patients (Supplemental Figure 1), both factors that likely contribute to the poor performance.

**Table 4:** Evaluation results.

			**TPR**			**PPV**			**F1**	
**Patient**	**True somatic**	**Filt**	**Pool**	**Joint**	**Filt**	**Pool**	**Joint**	**Filt**	**Pool**	**Joint**
GBM-003	256	0.95	0.65	0.61	0.34	0.81	0.91	0.50	0.72	0.73
GBM-005	179	1.00	0.66	0.87	0.22	0.80	0.94	0.36	0.72	0.90
GBM-006	150	0.85	0.45	0.61	0.16	0.70	0.83	0.27	0.54	0.70
GBM-008	212	1.00	0.76	0.83	0.31	0.83	0.96	0.47	0.80	0.89
GBM-009	179	0.99	0.77	0.81	0.25	0.72	0.95	0.40	0.74	0.88
GBM-014	285	0.90	0.52	0.44	0.36	0.77	0.73	0.52	0.62	0.55
GBM-016	301	0.85	0.70	0.77	0.30	0.84	0.91	0.44	0.77	0.84

*The number of true somatic variants found in each patient, as well as the sensitivity (TPR), precision (PPV), and F1 score are shown for the filtering (Filt), single sample (Pool), and lumosVar 2.0 (Joint) approaches*.

### Application to Archival Samples

**Figure 6:**
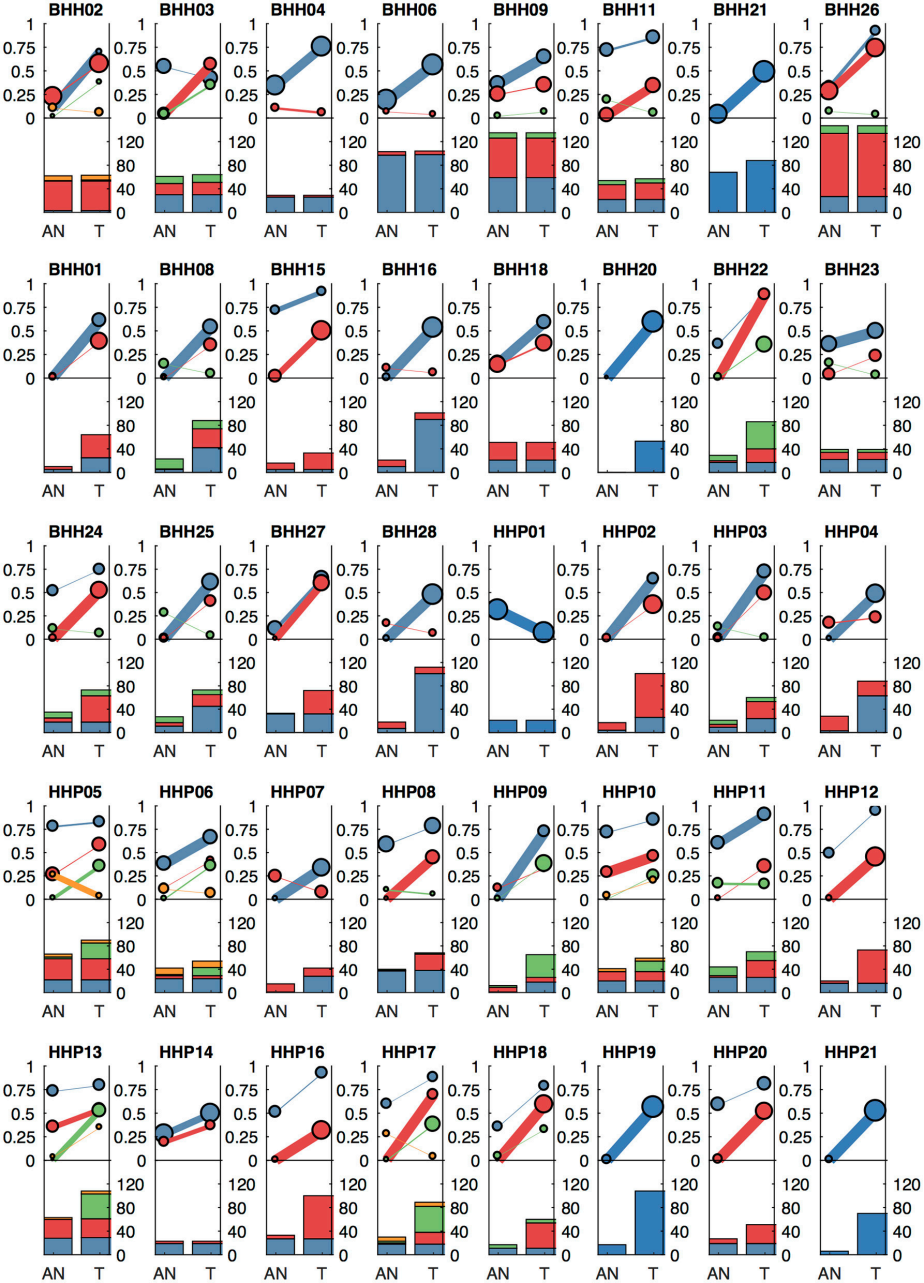
Clonal patterns and variant counts detected by lumosVar 2.0 in the archival dataset. The top half of each plot shows the summary of the clonal variant group patterns for each patient. Each line represents a clonal variant group and the thickness of the lines represents the proportion of copy number events assigned to each group and the size of each circle is proportional to the number mutations assigned to each group. The bottom half of each plot shows the number of somatic variants detected in the adjacent normal (AN) and tumor (T) samples, with the colors corresponding the clonal variant groups. The 8 patients in the top row had the adjacent normal tissue macrodissected from tumor containing slides and these patients typically have similar number of variants detected in the tumor and adjacent normal.

We applied our methods to archival breast cancer and prostate samples, where only FFPE tissue sections from biopsies or surgical tumor resections were available. For eight of the breast cancer patients, whole slides with adjacent histologically normal tissue were not available or did not have sufficient DNA yield, so adjacent normal areas were macro-dissected from tumor-containing slides. For the remaining patients, DNA was isolated from whole slides from additional FFPE blocks containing adjacent histologically normal tissue (Table 1). For two breast cancer cases (BHH02, BHH27), the additional “low tumor” blocks were from the contralateral breast following double mastectomy, though BHH02 was one of the eight patients that required macro-dissections of the tumor-containing slide to get sufficient DNA for the adjacent normal sequencing. Where macro-dissection was used to obtain the normal tissue samples, most of the somatic variants called were detected in the adjacent normal sample (median of 98%). For the patients where adjacent histologically normal tissue was obtained from separate slides, most patients still had some somatic variants detected in the normal tissue (median 35%–Figure 6).

**Figure 7:**
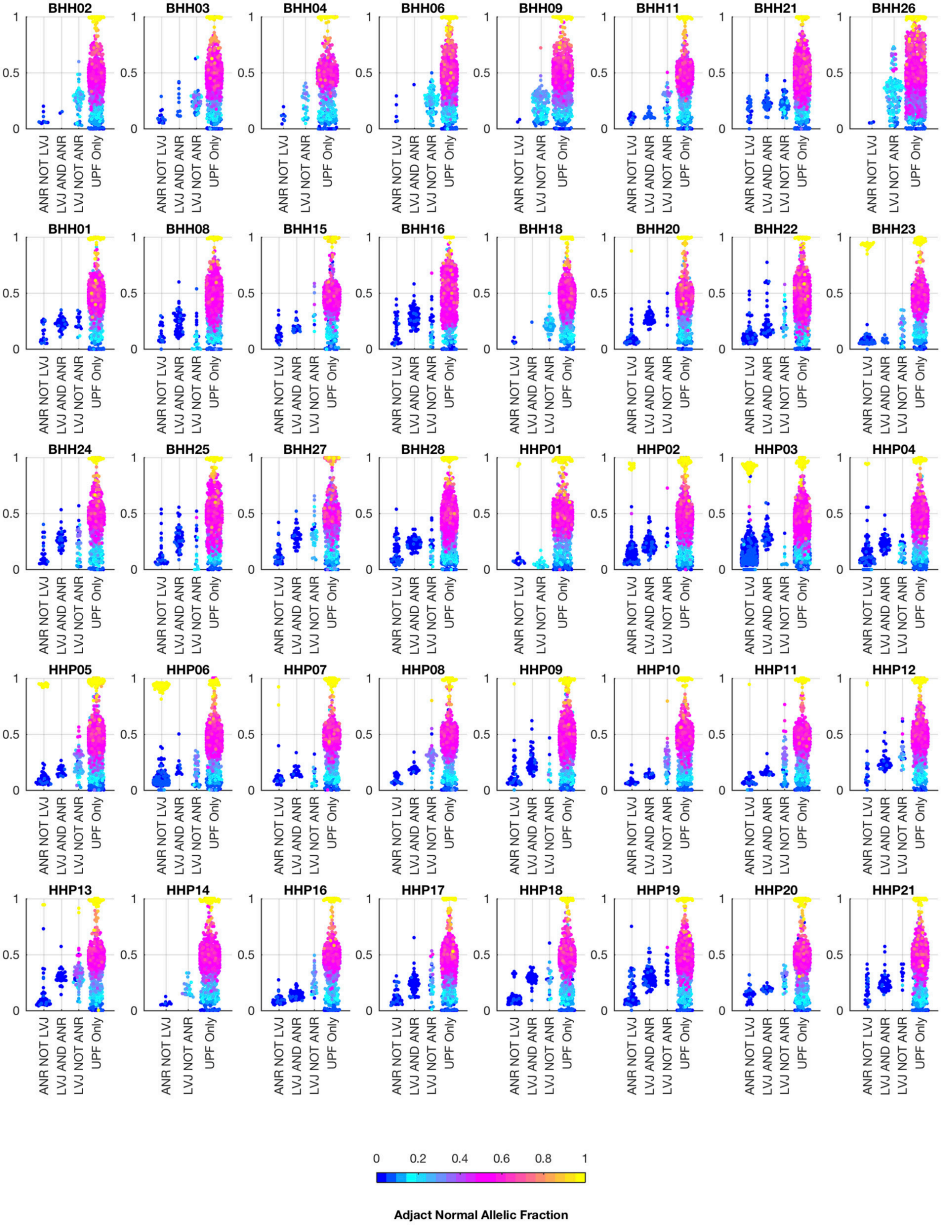
Comparison of allelic fractions of variants in archival dataset by calling method. For each of the breast and prostate patients, the allele fractions in the tumor sample are plotted for the variants detected in each of the three approaches. The color of each point indicates the allele fraction of the variant in the adjacent normal sample. Most of the variants detected in the adjacent normal as reference approach, but not lumosVar 2.0 joint analysis (ANR NOT LVJ), have low allele fractions in both the tumor and the adjacent normal. The variants detected by lumosVar 2.0 joint analysis, but not adjacent normal as reference approach (LVJ NOT ANR) typically have higher allele fractions in the tumor, and lower allele fractions in the adjacent normal, though lumosVar 2.0 joint analysis also detects some variants that are lower allele fraction in the tumor and higher allele fraction in the adjacent normal in a few patients such as HPP01. The variants only called in the unmatched filtering (UPF only) approach have similar allele fractions in the tumor and adjacent normal samples. The 8 patients in the top row had the adjacent normal tissue macrodissected from tumor containing slides and these patients typically have more variants detected by lumosVar 2.0 joint analysis and not ANR compared to the remaining patients whose adjacent normal sample was procured from separate slides.

We also analyzed the archival tissue using two additional approaches: (1) a filtering strategy where standard somatic variant calling tools were used against an unmatched reference (GM12878), and variants found in dbSNP were excluded as likely germline, referred to as the unmatched plus filtering approach (UPF), and (2) a strategy that used the tumor adjacent normal sample as the normal reference in standard somatic variant calling tools, referred to as the adjacent normal as reference approach (ANR). In both cases the same three paired somatic variant calling tools (mutect, seurat, and strelka) were used and variants were considered positive if they were called by at least two callers. While the adjacent normal tissue was selected based on histology, we do not expect it to be free of molecular alterations due to potential contamination, field cancerization, or tissue specific mutational processes. We include the ANR strategy for comparison, as it is a commonly used strategy when other constitutional tissue is not available (9). Using the UPF strategy, we found that most of the variants called using the filtering strategy have variant allele fractions around 50% in both the low- and high-tumor-content samples, suggesting that most are private germline variants. Using the ANR approach, we only identified variants with allele fractions in the adjacent normal sample that were at or very close to zero. The variants called by lumosVar 2.0 generally have higher allele fractions in the tumor samples and low allele fractions in the adjacent normal samples, as expected (Figure 7).

In order to compare the ability of the three approaches to detect likely drivers, mutations called by any of the three approaches were compared against the Cancer Hotspots database, which reports recurrent mutations in 11,119 tumor samples (Cancer Hotspots^1^). A total of 28 hotspot mutations were called including eight mutations with *in vitro* or *in vivo* validation (level-3), two mutations detected in the Cancer Hotspots dataset that were previously reported (level-2), and eighteen mutations that were novel in the Cancer Hotspots dataset (level-1). Of the ten level-3 and level-2 mutations, all were called in the UPF approach, lumosVar 2.0 joint analysis called eight, and only six were called in the ANR approach (Table 5). The two level-2 and level-3 mutations missed by lumosVar 2.0 had low allele fractions in the tumor sample (5–6%) and were not detected in the adjacent tissue, while the four level-3 hotspots variants missed by the ANR approach had moderate allele fraction in the tumor (17–35%) and low allele fractions in the adjacent tissue (2–16%). Seventeen of the eighteen level-1 hotspots were called only in the UPF approach, and these tended to have similar allele fractions in the tumor and adjacent normal samples. These include the same APOBR mutation called in 13 patients, and the same DHRS4 mutation called in four patients (Supplemental Table 1). Putative mutations that are common within a dataset, but not known to be common in cancer, are suggestive of alignment artifacts (26). Both the UPF approach and lumosVar 2.0 detected the eighteenth level-1 hotspot which was a CDH3 truncating mutations with high allele fractions in the tumor and low in the adjacent normal.

**Table 5:** Hotspot mutation detection.

**Patient**	**Gene**	**AA change**	**AD AN (AF)^a^**	**AD TM (AF)^b^**	**LVJ**	**ANR**	**CHL^c^**	**Cosmic count**
BHH01	PIK3CA	H1047R	473,9 (0.02)	378,201 (0.35)	Yes	LQC^d^	3	1,806
HHP13	PIK3CA	E545K	332,0 (0)	195,11 (0.05)	No	Yes	3	332
BHH06	AKT1	E17K	121,23 (0.16)	151,62 (0.29)	Yes	No	3	295
BHH24	AKT1	E17K	123,1 (0.01)	118,60 (0.34)	Yes	Yes	3	295
BHH28	PIK3CA	H1047L	417,0 (0)	348,81 (0.19)	Yes	Yes	3	262
HHP19	TP53	G245S	186,1 (0.01)	61,76 (0.55)	Yes	Yes	3	81
BHH18	PIK3CA	Q546E	201,16 (0.07)	164,38 (0.19)	Yes	No	3	3
BHH18	PIK3CA	G106R	127,8 (0.06)	96,19 (0.17)	Yes	No	3	2
BHH25	PIK3CA	E726K	269,0 (0)	268,17 (0.06)	No	Yes	2	31
BHH25	SF3B1	K666E	210,0 (0)	122,46 (0.27)	Yes	Yes	2	19

a *Allelic depth of reads supporting the reference, alternate alleles in the adjacent normal sample*.

b *Tumor sample*.

c *Cancer hotspots database validation level (Cancer Hotspots)*.

d *Called by one of three paired somatic variant callers (strelka)*.

## Discussion

Detecting somatic mutations when a normal tissue sample is not available remains a challenging problem. We present a method that leverages tumor-adjacent normal tissue, and is robust to significant levels of tumor contamination. Simulation studies suggest that a multi-sample approach should be more powerful than a single-sample approach, even if there is a small difference in tumor content between the two samples. Evaluation of a set of GBM samples with low tumor content (from NE biopsies) and high tumor content (from CE resections) further demonstrates the sensitivity and precision of the joint approach. Practical application of this approach to a set of FFPE breast and prostate samples shows the feasibility of this approach with typical archival samples.

While the approach described here represents an improvement over other tumor only somatic variant calling approaches, we believe it is best to sequence a true constitutional sample when feasible as the sensitivity of our approach is still limited compared to standard paired somatic variant calling. However, there are many open questions in precision oncology that can only be answered by collecting large amounts of patient genomic data linked to treatment response and clinical outcomes. For example, many factors may contribute to patient response to a targeted therapy, including the presence of other aberrations affecting the same pathway, aberrations affecting alternative pathways, and sub-clonal resistance mutations. Banks of archival samples show great potential to accelerate research predicting treatment response, as medium- and long-term outcomes may already be known. The approach outlined here should enable researchers to use archival samples more effectively, as accurately calling somatic variants is the first step in any analysis to answer such critical questions.

A complex relationship exists between tumor heterogeneity and clinical outcome, with moderately heterogeneous tumors having worse outcomes than both more homogenous tumors and more heterogeneous tumors (27, 28). Measuring the overall level of heterogeneity, in addition to detecting the clonal prevalence of individual variants, can provide insight into susceptibility and resistance to targeted therapies (29). lumosVar 2.0's ability to jointly analyze multiple samples from the same patient and integrate copy number and mutation data should be useful even when a matched normal sample is available to track mutations across longitudinal sample collections and spatially diverse samples, to gain insight into the tumor's evolution. Future work will further evaluate and benchmark lumosVar 2.0's clonal variant group analysis.

Compared to the single sample lumosVar 1.0 analysis, the joint approach requires lower total sequencing coverage to obtain the same sensitivity. Based on the simulation studies, we find that if the adjacent normal tissue has <25% tumor cell contamination, and the tumor sample has at least 55% percent tumor cells, then only 200X total coverage (100X for each sample) is required to detect 80% of the somatic variants that are in all of the tumor cells. However, higher coverage would be desirable in order to detect low abundance sub-clonal variants. We have shown that lumosVar 2.0 works best with a high tumor content and low tumor content sample from the same patient. These may not always be available such as with fine needle biopsies, or with brain tumors or metastases where resection of adjacent normal tissue would be avoided. However, we believe that the breast and prostate tumor blocks used in this study represent fairly typically archival samples, demonstrating the utility of the approach. Due to the large difference in prior probabilities of homozygous reference vs. somatic variants in this model, lumosVar 2.0 tends to be less sensitive to low abundance variants compared to other somatic variant callers. lumosVar 2.0 also has more stringent quality filtering than most paired somatic variant callers because the same artifacts often appear in the tumor and germline sample, so paired callers can use the presence in the germline to eliminate those artifacts. The probability that a variant is somatic or germline is calculated assuming that the allele specific copy number of the position is known with certainty, while there is clearly uncertainty in both setting the segmentation boundaries assigning both the copy number state of a given segment. Inspection of incorrectly classified variants suggests that segmentation boundary placement is a major source of error. We believe that a more sophisticated segmentation algorithm that is able to capture the uncertainty of segmentation boundary placement would yield the largest improvements in performance. We also recognize that an underlying assumption of our copy number model, that at most one copy number altered state may occur in a given segment across the patient samples, is an oversimplification, and a more realistic copy number model may improve both the copy number and variant calling results.

Though it may seem surprising that somatic variants were detected in histologically normal tumor adjacent tissue, previous studies have identified DNA, epigenetic, and gene expression alterations in tumor adjacent tissue (30). The theory of field cancerization proposes that epigenetic changes in the adjacent tissue creates a permissive environment for malignant transformation and sometimes can lead to multifocal disease and/or clonally independent recurrence. The sequencing of DNA from tumor adjacent tissue could serve a dual purpose in helping to identify somatic mutations when another source of normal tissue is not available, as well as helping to better understand the phenomenon of field cancerization.

## Data Availability

The glioblastoma evaluation dataset is being submitted to dbGap under the accession phs001460.v1.p1 (https://www.ncbi.nlm.nih.gov/projects/gap/cgi-bin/study.cgi?study_id=phs001460.v1.p1).

## Ethics Statement

This study was carried out in accordance with the recommendations of HHS regulations, 45 CFR part 46 and HonorHealth with written informed consent from all breast cancer participants with waiver of consent for prostate cancer participants. All breast cancer participants gave written informed consent in accordance with the Declaration of Helsinki. The protocol was approved by the Western Institutional Review Board (WIRB protocol #20161603).

## Author Contributions

RH designed and implemented the lumosVar 2.0 software. SiK designed and implemented the custom pileup engine. RH, ET, JA, WL, NH, JN, CK, RK, MB, and SB were involved in designing and generating data for the breast and prostate study. RH, NT, MB, and SB were involved in the analysis and interpretation of the GBM study. DE assisted with data analysis. SeK aided in mathematical formulations. RH, SeK, and SB drafted and edited the manuscript. All authors have read and approved the manuscript.

### Conflict of Interest Statement

RK and NH were employed by company Imaging Endpoints. The remaining authors declare that the research was conducted in the absence of any commercial or financial relationships that could be construed as a potential conflict of interest.

## Footnotes

^1^Available at: http://cancerhotspots.org/#/home [Accessed December 15, 2017]

## Supplementary Material

Supplemental Figure 1 lumosVar 2.0 output for GBM-014. Plots are analogous to Figure 4.

Supplemental Table 1 Level One Hotspot Variants.
