# Supplementary figures and images for: Leveraging Spatial Variation in Tumor Purity for Improved Somatic Variant Calling of Archival Tumor Only Samples
Source: Front Oncol. 2019 Mar 20;9:119. doi: 10.3389/fonc.2019.00119 (PMC6435595; doi:10.3389/fonc.2019.00119)

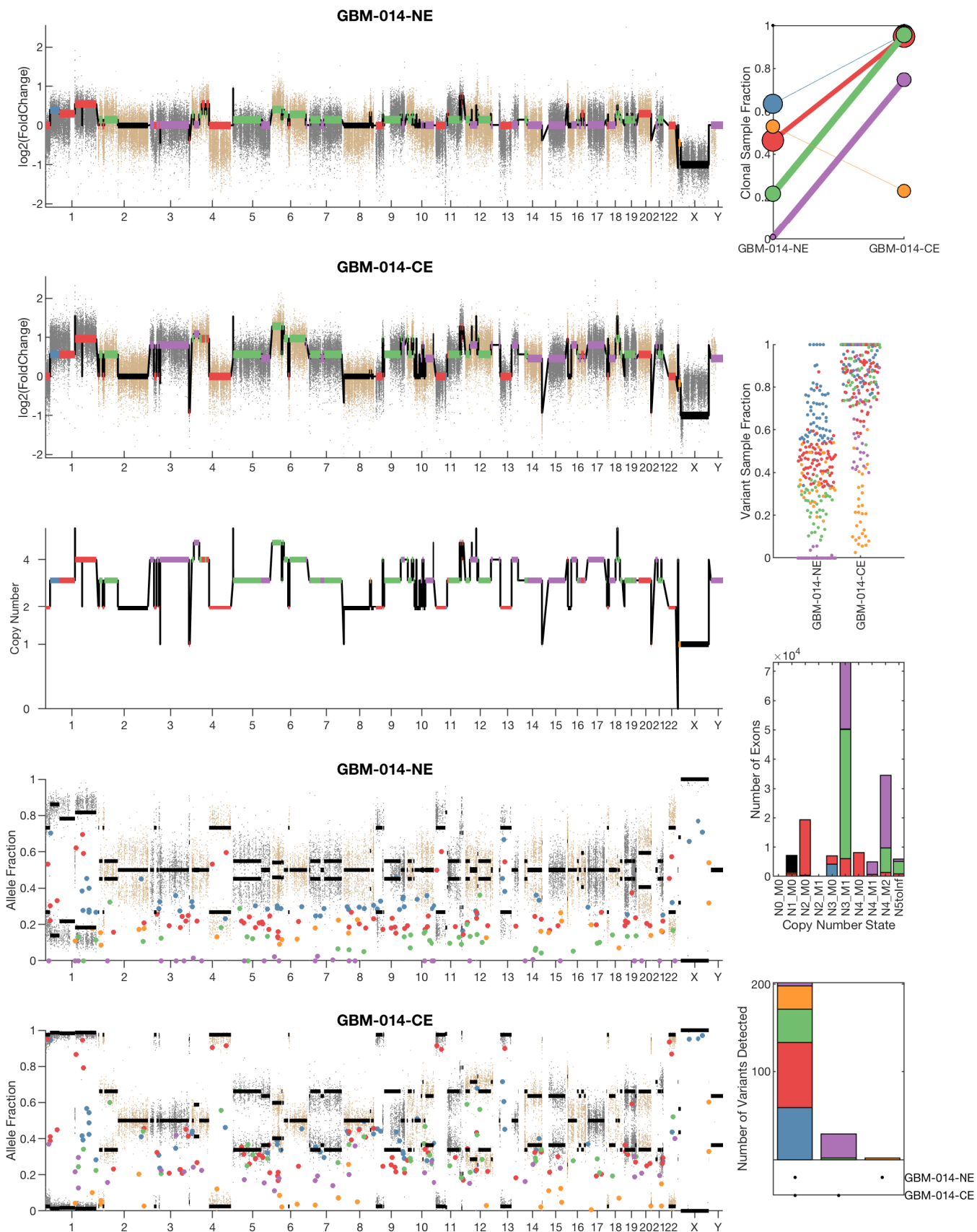

Supplement: Supplemental Figure 1 — lumosVar 2.0 output for GBM-014. Plots are analogous to Figure 4. [file Image_1.pdf]
