# Supplementary material for: Leveraging Spatial Variation in Tumor Purity for Improved Somatic Variant Calling of Archival Tumor Only Samples
Source: Front Oncol. 2019 Mar 20;9:119. doi: 10.3389/fonc.2019.00119 (PMC6435595; doi:10.3389/fonc.2019.00119)
Supplement: Supplemental Table 1 — Level One Hotspot Variants. [file Table_1.DOCX]

**Supplemental Table 1. Level One Hotspot Variants**

| **Patient** | **Gene** | **AA Change** | **AD AN (AF)** | **AD TM (AF)** | **LVM** | **ANR** | **CHL** | **Cosmic Count** |
| --- | --- | --- | --- | --- | --- | --- | --- | --- |
| BHH02 | APOBR | E361D | 84,8 (0.09) | 104,11 (0.1) | No | No | 1 | 12 |
| BHH03 | APOBR | E361D | 76,6 (0.07) | 103,15 (0.13) | No | No | 1 | 12 |
| BHH06 | APOBR | E361D | 98,7 (0.07) | 178,13 (0.07) | No | No | 1 | 12 |
| BHH08 | APOBR | E361D | 57,20 (0.26) | 61,20 (0.25) | No | No | 1 | 12 |
| BHH09 | APOBR | E361D | 71,31 (0.3) | 60,15 (0.2) | No | No | 1 | 12 |
| BHH11 | APOBR | E361D | 60,14 (0.19) | 53,13 (0.2) | No | No | 1 | 12 |
| BHH15 | APOBR | E361D | 21,2 (0.09) | 45,8 (0.15) | No | No | 1 | 12 |
| BHH20 | APOBR | E361D | 29,1 (0.03) | 88,7 (0.07) | No | No | 1 | 12 |
| HHP05 | APOBR | E361D | 19,8 (0.3) | 33,9 (0.21) | No | No | 1 | 12 |
| HHP12 | APOBR | E361D | 28,7 (0.2) | 32,15 (0.32) | No | No | 1 | 12 |
| HHP16 | APOBR | E361D | 67,8 (0.11) | 125,13 (0.09) | No | No | 1 | 12 |
| HHP18 | APOBR | E361D | 35,9 (0.2) | 44,9 (0.17) | No | No | 1 | 12 |
| HHP19 | APOBR | E361D | 34,7 (0.17) | 55,12 (0.18) | No | No | 1 | 12 |
| BHH26 | CDH1 | Q23* | 242,71 (0.23) | 79,201 (0.72) | Yes | No | 1 | 11 |
| BHH04 | DHRS4 | T102M | 112,4 (0.03) | 114,9 (0.07) | No | No | 1 | 8 |
| BHH09 | DHRS4 | T102M | 130,4 (0.03) | 105,7 (0.06) | No | No | 1 | 8 |
| HHP04 | DHRS4 | T102M | 127,3 (0.02) | 71,7 (0.09) | No | No | 1 | 8 |
| HHP12 | DHRS4 | T102M | 97,9 (0.08) | 94,10 (0.1) | No | No | 1 | 8 |
